# The Complete Chloroplast Genome Sequences of *Fritillaria* *ussuriensis* Maxim. and *Fritillaria cirrhosa* D. Don, and Comparative Analysis with Other *Fritillaria* Species

**DOI:** 10.3390/molecules22060982

**Published:** 2017-06-13

**Authors:** Inkyu Park, Wook Jin Kim, Sang-Min Yeo, Goya Choi, Young-Min Kang, Renzhe Piao, Byeong Cheol Moon

**Affiliations:** 1 K-Herb Research Center, Korea Institute of Oriental Medicine, Daejeon 305-811, Korea; pik6885@kiom.re.kr (I.P.); ukgene@kiom.re.kr (W.J.K.); yeosm1115@kiom.re.kr (S.-M.Y.); serparas@kiom.re.kr (G.C.); ymkang@kiom.re.kr (Y.-M.K.); 2 Department of Agronomy, Yanbian University Agriculture College, Yanji 133002, China; rzpiao@ybu.edu.cn

**Keywords:** *Fritillaria ussuriensis*, *Fritillaria cirrhosa*, chloroplast genome, comparative analysis, highly divergent region

## Abstract

The genus *Fritillaria* belongs to the widely distributed Liliaceae. The bulbs of *Fritillaria*, *F. ussuriensis* and *F. cirrhosa* are valuable herbaceous medicinal ingredients. However, they are still used indiscriminately in herbal medicine. Identification and molecular phylogenic analysis of *Fritillaria* species are therefore required. Here, we report the complete chloroplast (CP) genome sequences of *F. ussuriensis* and *F. cirrhosa*. The two *Fritillaria* CP genomes were 151,524 and 151,083 bp in length, respectively, and each included a pair of inverted repeated regions (52,678 and 52,156 bp) that was separated by a large single copy region (81,732 and 81,390 bp), and a small single copy region (17,114 and 17,537 bp). A total of 111 genes in *F. ussuriensis* and 112 in *F. cirrhosa* comprised 77 protein-coding regions in *F. ussuriensis* and 78 in *F. cirrhosa*, 30 transfer RNA (tRNA) genes, and four ribosomal RNA (rRNA) genes. The gene order, content, and orientation of the two *Fritillaria* CP genomes exhibited the general structure of flowering plants, and were similar to those of other *Fritillaria* species. Comparison of the six *Fritillaria* species’ CP genomes indicated seven highly divergent regions in intergenic spacers and in the *matK*, *rpoC1*, *rpoC2*, *ycf1*, *ycf2*, *ndhD*, and *ndhF* coding regions. We established the position of the six species through phylogenic analysis. The complete chloroplast genome sequences of the two *Fritillaria* species and a comparison study are useful genomic information for identifying and for studying the phylogenetic relationship among *Fritillaria* species within the Liliaceae.

## 1. Introduction

The genus *Fritillaria* belongs to the Liliaceae, which consists of 140 known species. The bulbs of several *Fritillaria* species (called “Pae-mo” in Korean and “Bei-mu” in Chinese) are important ingredients in herbal drugs that are used in oriental medicine, and have great economic value in Asian countries. *Fritillaria* species are distributed in temperate regions of the Northern hemisphere [1]. *Fritillaria* (*F.*) *cirrhosa* is mainly distributed in the alpine regions of Northwestern China (Gansu, Qinghai, Sichuan, Xizang, and Yunnan provinces) at altitudes of 3200–4600 m, whereas *F. ussuriensis* is distributed in the lowland regions (0–500 m altitude) of the northeastern and the far-eastern areas of China, Russia, and Korea [2]. *Fritillaria ussuriensis* and *F. cirrhosa* display morphological differences in their floral bud, bract, stem, and capsule [2]. The stem length of *F. ussuriensis* is 50–60 cm (maximum: 100 cm); the stem length of *F. cirrhosa* is less than 60 cm. *Fritillaria cirrhosa* has three bracts per flower, whereas *F. ussuriensis* has two. The tapals are yellow or yellowish green in *F. cirrhosa*; the petals are purple in *F. ussuriensis*. The capsules of *F. cirrhosa* are narrowly winged; the capsules of *F. ussuriensis* are wingless. While it is difficult to classify the plants according to the morphological characteristics of their bulbs, the flowering plants can be easily distinguished. The bulbs are divided into two and five types in the Korean Pharmacopoeia and the Chinese Pharmacopoeia, respectively [3,4]. The dried bulbs of *Fritillaria ussuriensis* Maxim. and *Fritillaria cirrhosa* D. Don are used in different herbal medicines—namely, Fritillariae Ussuriensis Bulbus (Ping-bei-mu in Chinese) and Fritillariae Cirrhosae Bulbus (Chuan-bei-mu in Chinese), respectively. Fritillariae Cirrhosae Bulbus and Fritillariae Ussuriensis Bulbus have separately been used for clinical purposes in traditional Korean medicine. In detail, the former has been used to treat cough due to deficiency of the lung, asthenia of the viscera, and tidal fever, and the latter has mainly been used to treat cough due to exogenous dryness and deficiency of Um (Yin; Chinese traditional medicine technical term). Furthermore, Fritillariae Cirrhosae Bulbus and Fritillariae Ussuriensis Bulbus have been prescribed to treat pulmonary carbuncle and bloody sputum, respectively [5]. Although the bulbs of *Fritillaria* have value in herbal medicine, different *Fritillaria* species are still used indiscriminately because of their morphological similarity and similar names [6]. Therefore, accurate identification of *Fritillaria* species (e.g., using molecular markers) is required to identify medicinal plants and the drugs derived from them [6,7].

Chloroplasts play an important role in photosynthesis and carbon fixation as well as in the biosynthesis of starch, fatty acids, and amino acids [8,9]. The chloroplast (CP) genome ranges from 120 to 180 kb in higher plants and has a quadripartite structure consisting of a large single copy (LSC) region, a small single copy (SSC) region, and two copies of a larger inverted repeat (IR) [10,11]. The CP genome encodes 110 to 130 genes with up to 80 unique protein-coding genes, four ribosomal RNAs (rRNAs), and approximately 30 transfer RNAs (tRNAs). However, few parasitic plants have small chloroplast genomes due to their unique life cycles [12]. Since the CP genome of *Marchantia polymorpha* [13] was reported in 1986, more than 500 complete chloroplast genome sequences have been deposited in the GenBank database [14]. With the advancement of next-generation sequencing (NGS) technology, chloroplast genome assembly has become cheaper and easier compared to the Sanger method [15]. Through the comparison of chloroplast genomes, the development of molecular markers has also become more cost effective. The CP genome has been widely used for understanding phylogenetic relationships and discovering useful molecular markers, which are used in DNA barcoding to identify plant species and authenticating and in identifying herbal medicines. In particular, *matK* and *rbcL* are used as universal plant DNA barcodes [16].

Here, we report the de novo assembly of *F. ussuriensis* and *F. cirrhosa* CP genomes using the Illumina platform. This is the first comparative analysis of *Fritillaria* CP genomes in conjunction with previously reported CP genomes. This study aims to investigate global structural patterns of six *Fritillaria* CP genomes and also to discover highly divergent regions among the species. We also analyzed phylogenic relationships among the six *Fritillaria* species. The results provide basic knowledge on characteristics of Fritillaria species and enhance our understanding of Fritillaria species evolution within the Liliaceae.

## 2. Materials and Methods 

### 2.1. Genome Sequencing and Assembly

Fresh leaves of *F. ussuriensis* (KY646166) and *F. cirrhosa* (KY646167) were collected from medicinal plant plantations, and the samples were used for CP genome sequencing. *Fritillaria ussuriensis* and *F. cirrhosa* were given identification numbers, and specimens were registered in the Korean Herbarium of Standard Herbal Resources (Index Herbariorum code KIOM) at the Korea Institute of Oriental Medicine (KIOM). The extraction of DNA was conducted with the DNeasy Plant Maxi kit (Qiagen, Valencia, CA, USA), according to the manufacturer’s instructions. Illumina short-insert paired-end sequencing libraries were constructed and generated using the NextSeq platform (Illumina, San Diego, Valencia, CA, USA) by LabGenomics, Korea. The CP genomes were obtained by the de novo assembly method from low-coverage whole-genome sequence derived from the Phyzen pipeline [17]. Trimmed paired-end reads (Phred scores ≥20) were assembled using CLC genome assembler (ver. 4.06 beta, CLC Inc, Rarhus, Denmark) with default parameters. SOAPdenovo gap closer was used to fill gaps based on alignment paired-end reads [18]. The principal contigs representing the CP genome were retrieved from the total contigs using Nucmer [19], and aligned contigs were ordered with the CP genome sequence of *Fritillaria hupehensis* (NC024736) [20].

### 2.2. Genome Annotation and Comparative Analysis

Gene annotation of *F. ussuriensis* and *F. cirrhosa* CP genomes was performed using DOGMA annotation [21], and manually corrected for codons and gene boundaries using BLAST searches. The tRNAs were confirmed with tRNAscan-SE 1.21 [22]. The circular maps of the two *Fritillaria* CP genomes were obtained using OGDRAW [23]. GC content and codons were analyzed using MEGA6 software [24]. The mVISTA program was used to compare the seven *Fritillaria* CP genomes using the *F. ussuriensis* CP genome as reference [25]. Five *Fritillaria* CP genomes were downloaded from GenBank (*F. hupenesis*: NC024736, *F. tapaiensis*: NC023247, *F. unibracteata*: KF769142, and *F. thunbergii*: KY646165).

### 2.3. Repeat Analysis 

SSRs in *F. ussuriensis* and *F. cirrhosa* CP genomes were detected using MISA [26] with the parameters set to minimum number of repeats: 10, 5, 4, 3, 3, and 3 for mono-, di-, tri-, tetra-, penta-, and hexa-nucleotides, respectively. The tandem repeats were 20 bp or more with minimum alignment score and maximum period size set at 50 and 500, respectively, and the identity of repeats was set to ≥90% [27]. IRs were detected using Inverted Repeat Finder with default parameters. The IRs were 20 bp or more with 90% similarity [28]. 

### 2.4. Phylogenic and Divergence Analysis

A molecular phylogenetic tree was constructed using 74 protein-coding genes from 11 species. Among these 11 taxa, nine completed CP genomes were downloaded from NCBI: *Fritillaria unibracteata* (KF769142), *Fritillaria taipaiensis* (NC023247), *Fritillaria hupehensis* (NC024736), *Fritillaria thunbergii* (KY646165), *Lilium superbum* (NC026787), *Lilium longiflorum* (KC968977), *Smilax china* (HM536959), *Acorus gramineus* (NC026299), and *Cocos nucifera* (KX028884). A total of 71 protein-coding genes were aligned with MAFFT [29]. Maximum likelihood (ML) and maximum parsimony (MP) analyses were performed using MEGA6 with 1000 bootstrap replicates [24]. Six *Fritillaria* species CP genomes were aligned using MAFFT, and the sequences were manually adjusted with Bioedit [30]. To calculate nucleotide variability (Pi) between CP genomes, sliding window analysis was performed using DnaSP version 5.1 software [31]. Window length was set to 600 bp, and the step size was 200 bp.

## 3. Results

### 3.1. Chloroplast Genome Organization of Two *Fritillaria* Species

**Figure 1:**
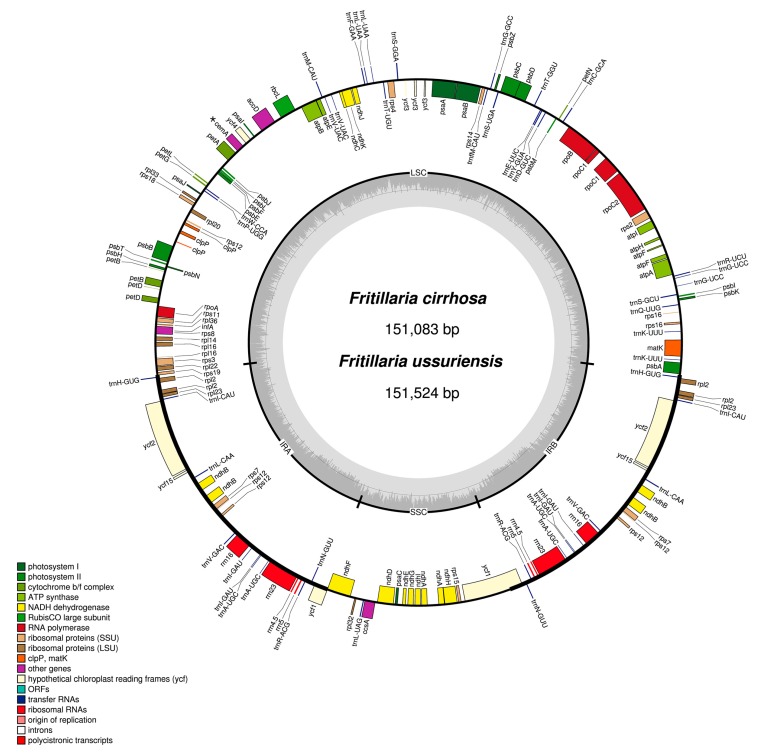
Circular gene map of chloroplast genomes of two *Fritillaria* species. Genes drawn inside the outer layer circle are transcribed clockwise, and those outside the circle are transcribed counterclockwise. The darker gray in the inner circle corresponds to GC content. * *cemA* is pseudogene in *F. ussuriensis*.

Illumina sequencing generated 5.0 and 4.5 Gb of trimmed paired-end reads from *Fritillaria ussuriensis* and *Fritillaria cirrhosa*, respectively. From the *de novo* assembly sequence that uses low-coverage whole-genome sequencing (WGS), we obtained ten and eight contigs covering the whole chloroplast genome sequences of *F. ussuriensis* and *F. cirrhosa*, respectively (Table S1). Single circular sequences were completed after gap filling and manual editing. The complete circular chloroplast genomes of *F. ussuriensis* and *F. cirrhosa* were 151,524 and 151,083 bp, with approximately 256× and 452× coverages, respectively (Table S2). Paired-end read mapping was conducted to validate the draft genome, which was compared to our draft genomes and the previously reported *F. hupehensis* genome using BLASTZ program (Figure S1). Both *F. ussuriensis* and *F. cirrhosa* chloroplast genomes had a quadripartite structure similar to most land plants consisting of a pair of IRs (52,678 and 52,156 bp, respectively). In addition, both *F. ussuriensis* and *F. cirrhosa* chloroplast genomes were also separated by large single copy (LSC; 81,732 and 81,390 bp) and small single copy (SSC; 17,114 and 17,537 bp) regions (Figure 1, Table 1). The *Fritillaria* chloroplast genomes were AT-rich (63% in both species), but the LSC (65.3% and 64.2% in *F. ussuriensis* and *F. cirrhosa*, respectively) and SSC (69.4% and 69.6% in *F. ussuriensis* and *F. cirrhosa*, respectively) regions were more AT-rich than the IR regions (57.6% and 57.4% in *F. ussuriensis* and *F. cirrhosa*, respectively), making the LSC and SSC regions more similar to other chloroplast genomes [10,32,33,34,35].

The gene content, order, and orientation were similar in *Fritillaria* CP genomes. There were 111 and 112 predicted genes in *F. ussuriensis* and *F. cirrhosa*, respectively. Of these, 94 in *F. ussuriensis* and 95 in *F. cirrhosa* were unique to the LSC and SSC regions, and 18 were duplicated in the IR regions (Table 1 and Table 2). The 111 and 112 unique genes consisted of 77 and 78 protein-coding regions in *F. ussuriensis* and *F. cirrhosa*, respectively. In the upstream region of the *F. ussuriensis* CP genome, one gene, *cemA*, had an internal stop codon (TGA). The gene *cemA* encodes a heme-binding protein in the chloroplast envelope membrane, which was lost in land plants due to the introduction of an internal stop codon. [8,10,36]. The two *Fritillaria* CP genomes had 30 tRNAs, with 17 duplicated genes including seven tRNAs (*trnA-UGC*, *trnI-CAU*, *trnI-GAU*, *trnL-CAA*, *trnN-GUU*, *trnR-ACG*, *trnV-GAC*), four rRNAs (*rrn16*, *rrn23*, *rrn4.5*, *rrn5*), and six protein-coding genes (*ndhB*, *rpl2*, *rpl23*, *rps12*, *ycf1*, *ycf2*). They also had 18 intron-containing genes, among which 15 (nine protein-coding genes and six tRNA genes) had a single intron and two genes (*ycf3* and *clpP*) had two introns each (Table S4). Thirteen genes (nine protein-coding and four tRNA genes) were located in the LSC region, a protein-coding gene in the SSC region, and four genes (two protein-coding and two tRNA genes) in the IR regions. The protein-coding genes included five genes (*ndhB*, *rpl2*, *rpl23*, *rps12*, *ycf2*) that were duplicated in the IR regions. The *rps12* gene was trans-spliced because the 5′ end was located in the LSC region and the 3′ end in the IR region. The *trnK-UUU* gene had the largest intron region (2613 bp in *F. ussuriensis* and 2562 bp in *F. cirrhosa*), including *matK.* The genes *psbT*, *rpl2*, and *ndhD* had the alternative start codon ACG, and *rps19* started with GTG. Use of ACG and GTG as start codons are common features of a variety of genes in the chloroplast genomes of land plants [37,38,39,40].

**Figure 2:**
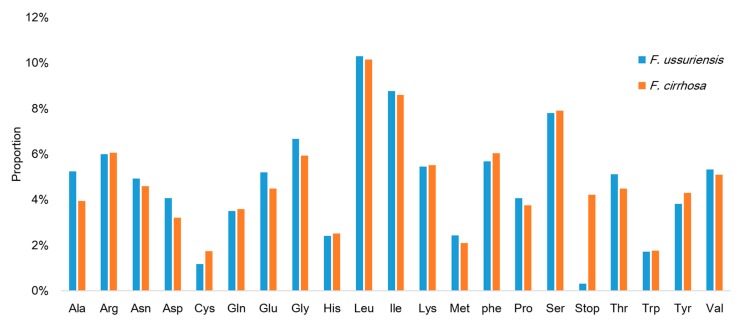
Amino acid frequencies in *Fritillaria (F.) ussuriensis* and *F. cirrhosa* protein-coding sequences.

Approximately 52% of *Fritillaria* chloroplast genomes consisted of protein-coding genes (78,951 bp in *F. ussuriensis* and 79,835 bp in *F. cirrhosa*), 1.9% of tRNAs (2876 bp in both species), and 6.0% of rRNAs (9048 bp in both species). The remaining 40.1% consisted of intergenic regions, non-coding introns, and pseudogenes. The *ycf1* gene located between the inverted repeat b (IRb) and the small single copy (SSC) region had premature stop codons in the coding sequence, and has been annotated as a pseudogene in other angiosperm chloroplast genomes [32,41,42,43]. The codon usage and anticodon recognition patterns of the CP genomes are summarized in Table S5. Protein-coding genes comprised 26,317 codons in *F. ussuriensis* and 26,611 in *F. cirrhosa*. Among these codons, those for leucine and isoleucine were the most common in both *Fritillaria* genomes, as observed previously in other land plant CP genomes (Figure 2) [10,44]. The 30 tRNA genes included codons for all 20 amino acids required for biosynthesis. Within protein-coding regions, the AT content for the first, second, and third codons were 55.1%, 62.1%, and 70.4% in *F. ussuriensis*, respectively, and 59.3%, 63.1%, and 65.6% in *F. cirrhosa*, respectively (Table S3). The bias towards a higher AT content at the third position has been observed in other land plant CP genomes [45,46,47].

### 3.2. Repeat Analysis in Two Fritillaria Chloroplast Genomes

**Figure 3:**
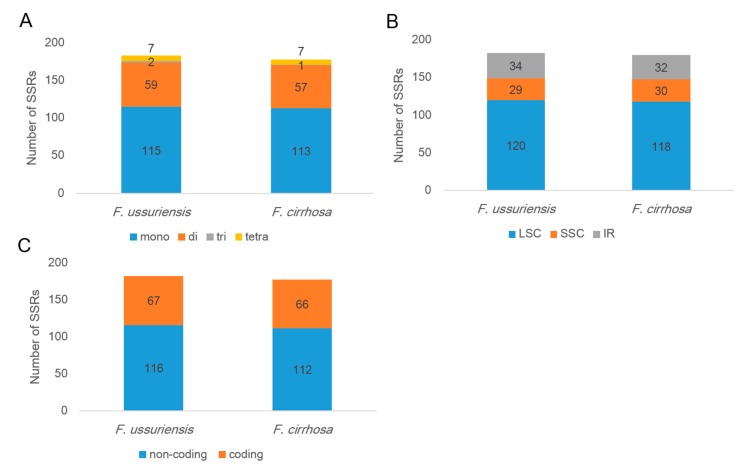
Distribution of SSRs (simple sequence repeat) in the *Fritillaria (F.) ussuriensis* and *F. cirrhosa* chloroplast (CP) genomes. (**A**) SSR type distribution in the two *Fritillaria* CP genomes; (**B**) The proportion of SSRs in different genomic regions of *Fritillaria* CP genomes; (**C**) SSR distribution between coding and non-coding regions.

Simple sequence repeats (SSRs) or microsatellites are tandem repeat sequences consisting of 1–6 nt sequence motifs in prokaryotic and eukaryotic genomes [48,49]. SSRs were detected using a microsatellite identification tool—MISA—in both *Fritillaria* CP genomes. We detected 183 and 178 SSRs in *F. ussuriensis* and *F. cirrhosa* CP genomes, respectively. Mononucleotide motifs were the most abundant type of repeat, and di-nucleotides were the second most abundant in both of the *Fritillaria* CP genomes (Figure 3). Almost all SSR loci were composed of A or T, which contributed to the bias in base composition (A/T; 63%) in both *Fritillaria* CP genomes. Simple sequence repeats (SSRs) were more abundant in non-coding regions than in coding regions, as evidenced by the presence of 63% of all SSRs in the non-coding regions of both genomes. Furthermore, most SSRs were located in the LSC region. In addition, 48 polymorphic SSRs were detected between the two *Fritillaria* CP genomes (Table S8). Most SSRs were located in the intergenic region with the A or the T motif. The longest polymorphic SSR was found in the *psbZ-trnG* region, and had a 12 bp length difference.

We also identified 15 tandem repeats in *F. ussuriensis* and 13 tandem repeats in *F. cirrhosa* of more than 20 bp (Table S6). Of these, most were located in IGS, LSC, and IR regions. The longest tandem repeats were 108 bp in *F. ussuriensis* (located in the *trnT-UGU*/*trnL-UAA* IGS) and 94 bp in *F. cirrhosa* (located in the *trnG-UCC*/*trnR-UCU* IGS). Four (two in IGS, two in CDS) tandem repeats represented the same region in both CP genomes. Four and six palindromic repeats were also detected in *F. ussuriensis* and *F. cirrhosa*, respectively (Table S7). In *F. ussuriensis*, two of these were located in the LSC region and the other two were located in the IR regions. Both species had palindromic repeats at four locations—namely, the IGS of *accD*/*psaI*, *petD*/*rpoA*, *ccsA*/*ndhD*, and *rps15*/*ycf1* regions.

### 3.3. Comparison of the Chloroplast Genomes with Those of Other *Fritillaria* Species

**Figure 4:**
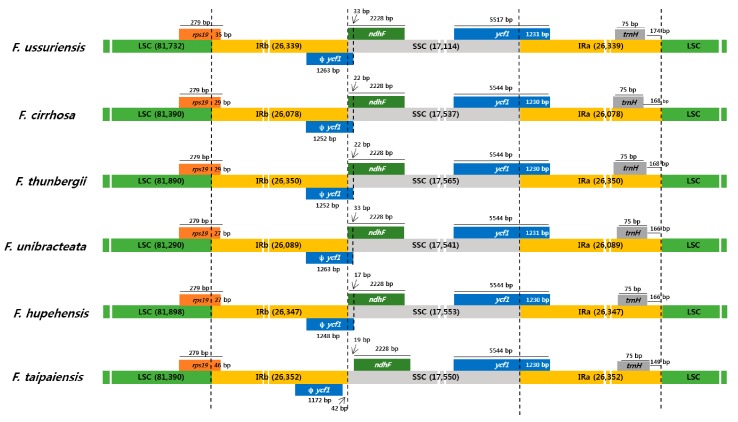
Comparison of large single copy (LSC), small single copy (SSC), and inverted repeat (IR) border regions among the six *Fritillaria* species’ chloroplast genomes. Colored boxes for genes represent the gene position. ψ: pseudogenes.

The two *Fritillaria* chloroplast genomes had approximately 98% sequence identity, and their gene content and order and genome structure were similar. The CP genome of *F. ussuriensis* was approximately 441 bp longer than *F. cirrhosa* (Table 1). The LSC and IR regions of *F. ussuriensis* were 342 bp and 522 bp longer, respectively, than *F. cirrhosa*. The SSC region of *F. ussuriensis* was 423 bp shorter than *F. cirrhosa*. IR contraction and expansion are common evolutionary events and contribute to genome size variation [10,32,50]. We analyzed the border structure of *F. ussuriensis* and *F. cirrhosa* genomes. Detailed comparison of the LSC, SSC, and IR regions are shown in Figure 4. The *rps19* gene located in the LSC region extended into the IRb region by 27–46 bp. The border between IRb/SSC and SSC/IRa extended into the *ycf1* genes if all *Fritillaria* species except for *F. taipaiensis*. Overlaps of 17–33 bp were observed between the *ycf1* pseudogene and *ndhF* gene, except in *F. taipaiensis*. The *trnH* genes were all located in the IR region, 149–174 bp away from the IRa/LSC boundary. The locations of most of the other genes (e.g., *ndhF* and *trnH*) were similar in both CP genomes (Figure 4).

**Figure 5:**
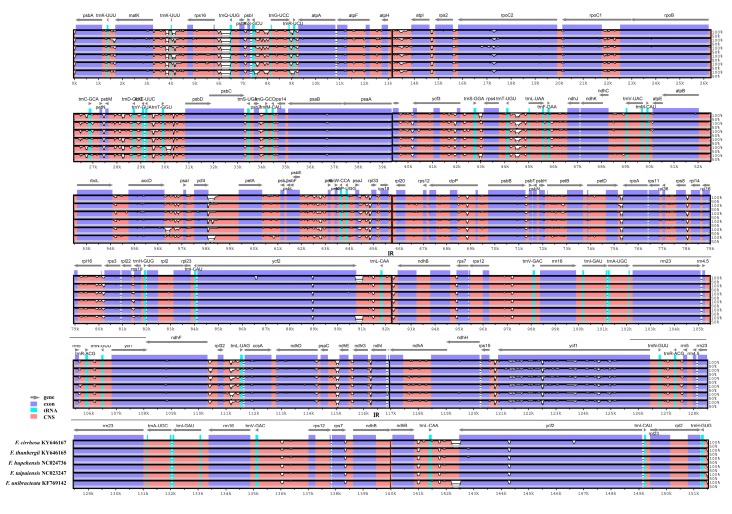
Comparison of six *Fritillaria* chloroplast genomes using mVISTA alignment program. The complete chloroplast (CP) genomes of six *Fritillaria* species were used for comparisons with published CP genomes. Blue block: conserved gene; sky-blue block: transfer RNA (tRNA) and ribosomal RNA (rRNA); red block: conserved non-coding sequences (CNS). White peaks indicate regions with sequence variation among *Fritillaria* species.

**Figure 6:**
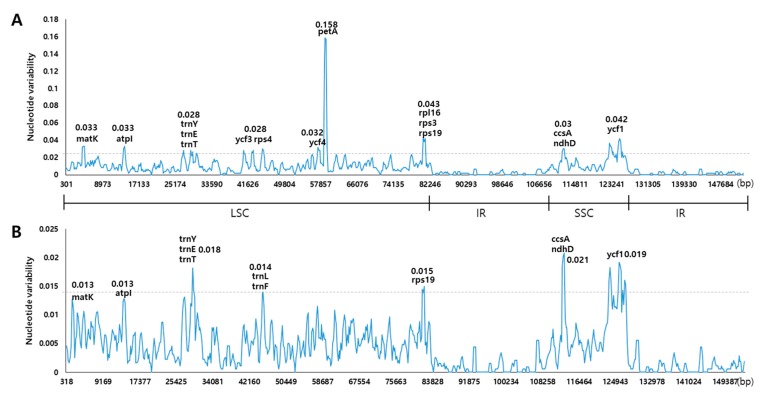
Sliding window analysis of the chloroplast (CP) genomes. (**A**) Comparison of the nucleotide variability (Pi) between *Fritillaria (F.) ussuriensis* and *F. cirrhosa*. (**B**) Comparison of the nucleotide variability (Pi) among six *Fritillaria* species’ cp genomes.

We performed multiple sequence alignments between six *Fritillaria* chloroplast genomes using mVISTA (Figure 5). The non-coding regions were more divergent than the coding regions. The most divergent regions were found in IGSs such as *matK*/*trnK-UUU*, *trnK-UUU*/*rps16*, *rps16*/*trnQ-UUG*, *psbK*/*psbI*, *atpH*/*atpI*, *psbM*/*trnD-GUC*, and *ycf4/petD*. For the coding regions, the most divergent regions included *matK*, *rpoC1*, *rpoC2*, *ycf1*, *ycf2*, *ndhD*, and *ndhF*. Previous studies reported similar divergent regions [32]. These regions are conserved regions with clusters of sequence variability called hotspots, containing single-nucleotide polymorphisms (SNPs) and indels [51,52]. The nucleotide variability—Pi—was calculated to show divergence at the sequence level of *Fritillaria* CP genomes. As expected, the IR regions were more conserved than the LSC and SSC regions. Between *F. ussuriensis* and *F. cirrhosa* CP genomes, Pi values (%) ranged from 0 to 15.8% with a mean of 0.9%. Ten highly divergent loci included *matK*, *atpI*, *trnY-GUA*, *trnE-UUC*, *trnT*, *ycf3*, *rps4*, *ycf4*, *petA*, *rpl16*, *rps3*, *rps19*, *ccsA*, *ndhD*, and *ycf1* (Figure 6). The most divergent region in the *ycf4*-*petA* (IGS) region showed 15.8% sequence variability. In the coding region, *rpl16* showed the highest degree of nucleotide variability (4.3%). The ten loci had a much higher sequence divergence value than the other regions (Pi > 2.7%). Among the six *Fritillaria* CP genomes, Pi values varied from 0 to 2.1% with a mean of 0.4% (Figure 6). The loci *matK*, *atpI*, *trnY*, *trnE*, *trnT*, *rps19*, *ccsA*, *ndhD*, and *ycf1* were highly divergent among *Fritillaria* species. The highest degree of variability was found in *ndhD* (2%).

### 3.4. Phylogenic Analysis

**Figure 7:**
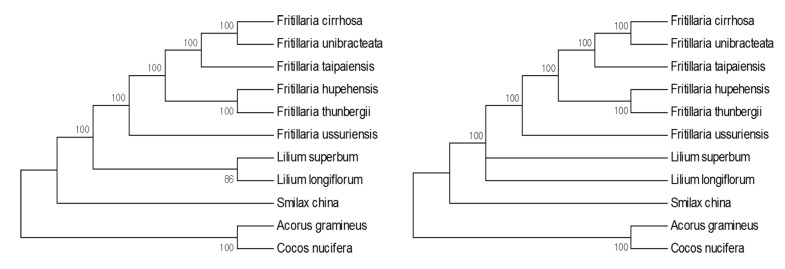
Phylogenetic trees constructed with 74 protein-coding genes of 11 species using maximum likelihood (ML) and maximum parsimony (MP) methods. Numbers above are bootstrap support values (>50%).

Chloroplast genome sequences have been successfully used in numerous phylogenetic studies of angiosperms [53,54]. To identify the phylogenetic position of the six *Fritillaria* species within the Liliaceae, 74 protein-coding sequences shared by 11 CP genomes were aligned (Figure 7). Two species, *Acorus gramineus* and *Cocos nucifera*, were set as outgroups. The alignment covered 80,532 bp. Maximum likelihood (ML) and maximum parsimony (MP) analyses revealed that six out of eight nodes had 100% bootstrap values. Both the ML and MP phylogenetic results strongly indicated that the genera—*Fritillaria* and *Lilium*—were clearly separated, and the six *Fritillaria* species were closely related within Liliaceae. *Fritillaria cirrhosa* and *F. unibracteata* formed a cluster. Subsequently, a monophyletic clade formed a cluster with *F. taipaiensis*, *F. hupehensis*, and *F. thunbergii*, which were related to *F. ussuriensis* as a monophyletic branch.

## 4. Discussion

Advances in NGS technologies make it possible to complete the entire chloroplast genome with the discovery of molecular markers [55,56]. We used low-coverage WGS data to obtain chloroplast genomes—an approach that has been successfully used in several other studies [17,57,58]. This approach requires less time and has a lower cost than the previously used method. Here, we obtained two *Fritillaria* chloroplast genomes and applied comparative analysis to six *Fritillaria* species CP genomes. The two *Fritillaria* CP genomes contained a pair of IRs, and LSC and SSC regions. The two *Fritillaria* genomes have similar genome structures, gene orders, and gene contents, including introns and base compositions. They show characteristics that are typical of land plant CP genomes [8,32].

SSRs are suitable molecular markers because they are distributed throughout the whole genome and display high polymorphism between species, locus-specific co-dominance, and high transferability [59]. SSRs play an important role in CP genome rearrangement during evolution [41,60]. The repeat units—A and T—appear most frequently in SSRs, which contribute to the AT-richness of the CP genome [61,62,63]. Recently, plastid genomic information obtained by NGS analysis has provided a resource for high-throughput screening of SSR loci and permitted efficient SSR marker development in various plant taxa [64,65]. In this study, we identified 183 and 178 SSR loci in *F. ussuriensis* and *F. cirrhosa* CP genomes, respectively (Figure 3). In addition, we also identified 48 potential polymorphic SSRs between the two *Fritillaria* species, *F. cirrhosa* and *F. ussuriensis* (Table S8). These abundant SSR loci and potential inter-species specific polymorphic SSRs could provide useful genetic information and sequence resources for further molecular genetic studies of *Fritillaria* species, including species identification, assessment of genetic diversity, ecological genetic studies, and evolution studies [66].

IR regions are the most conserved regions in the chloroplast genome [9]. The contraction and expansion at the borders of IR regions, however, are common evolutionary events, and they are the major causes of rearrangements and size variations [10,34,50]. In this study, we compared the SC/IR boundaries among six *Fritillaria* genomes. The SC/IR boundaries showed only slight differences, for example, at *ycf1* and *ѱycf1* (Figure 4). This phenomenon is relatively common in other CP genomes [35,41,67]. Several genes, including *ndhF*, *rps19*, and *trnH*, had almost identical locations and sizes among *Fritillaria*. Multiple sequences of alignment between the six *Fritillaria* genomes indicated that the IR regions were more conserved than the LSC and SSC regions due to copy corrections by gene conversion in the IR regions [68]. The most divergent regions were found in the IGS, which have been used in phylogenetic studies [11,69]. For the coding regions, the most divergent regions included *matK*, *rpoC1*, *rpoC2*, *ycf1*, *ycf2*, *ndhD*, and *ndhF*. Previous studies reported similar divergent regions [9]. These regions are conserved regions of clustered variation called hotspots, containing SNPs and indels [51,52]. The *rpoC2*, *rpoC1*, and *ycf1* genes are known to contain hotspots of sequence variation [11,51,52,70]. Therefore, as in other land plants, *Fritillaria* CP genomes contain general hotspot regions for genetic variation.

Chloroplast genomes provide an abundant genomic resource for phylogenetic analysis, and many studies have used protein-coding sequences or whole chloroplast genome sequences in these analyses [32,69,71]. In the present study, we conducted phylogenic analysis on the six *Fritillaria* species and found that the six species were well clustered according to the type of herbal medicine (Figure 7). Furthermore, *F. ussuriensis* could be clearly separated from other *Fritillaria* species, as previously reported [20,72]. In this study, phylogenetic analysis revealed clear positons of *Fritillaria* at the species level with 100% bootstrap values. *Fritillaria hupehensis* and *F. thunbergii* formed monophyly with 100% bootstrap values. Another monophyletic group clustered *F. cirrhosa* and *F. unibracteata* with *F. taipaiensis*. *Fritillaria ussuriensis* showed a distant genetic relationship to the other five *Fritillaria* species. In a previous study, a phylogenetic tree was constructed to verify the relationship between ninety-two *Fritillaria* species using a combined chloroplast region consisting of *matK*, *rbcL*, and *rpl16*. Ninety-two *Fritillaria* species were classified into eight *Fritillaria* subgenera while considering their geographic distributions [1]. *Fritillaria* thunbergii, *F. cirrhosa*, and *F. ussuriensis* were well classified into the *Fritillaria* subgenus—consistent with our results. Although the phylogenetic tree comprised only a few *Fritillaria* species, our results are well supported by the phylogenetic relationships of *Fritillaria* species reported previously [1]. Thus, phylogenetic analysis of CP genome sequences could provide useful information for uncovering relationships among *Fritillaria* species—in particular, details of their positions at the species level with 100% bootstrap values. However, the results suggested that additional large-scale genomic analyses using numerous accurately identified *Fritillaria* species are required to clarify the taxonomy and phylogenetic relationships of *Fritillaria* species at low taxonomic levels.

Molecular genetic tools using complete CP genome sequences provide an efficient and accurate way to authenticate herbal medicines. Universal DNA barcoding is widely used as a reliable genetic tool for identifying plant species; however, these methods are still limited in their ability to identify and discriminate medicinal plants at the species level for several taxa. The complete CP genome sequences could be useful for identifying plant species solely as a super DNA barcode, which provide high resolution at lower taxonomic levels [73]. In addition, the two complete *Fritillaria* CP genome sequences determined in this study could provide useful basic genomic information along with the previously reported *Fritillaria* species for studying phylogeny. The sequence variability from comparative analyses could be used as potential resources for further development of DNA molecular markers such as SSR or microsatellite marker. These additional developments could be used to study evolutionary properties and/or population genetics of *Fritillaria* species as well as DNA barcodes or SCAR markers.

## Figures and Tables

**Table 1:** Size comparison of two *Fritillaria* species’ chloroplast genomic regions.

Species	*Fritillaria ussuriensis*	*Fritillaria cirrhosa*
Total CP genome size (bp)	151,524	151,083
LSC (bp)	81,732	81,390
IR (bp)	52,678	52,156
SSC (bp)	17,114	17,537
GC content (%)	36.95	36.96
LSC (%)	34.71	34.79
IR (%)	42.40	42.60
SSC (%)	30.63	30.50
Total number of genes	111	112
Protein-coding gene	77	78
rRNA	4	4
tRNA	30	30

CP: Chloroplast; LSC: Large single copy; IR: Inverted repeat; SSC: Small single copy.

**Table 2:** Genes present in the two *Fritillaria* chloroplast genomes.

Gene Products of Two *Fritillaria* Species	
Photosystem I	*psaA*, *B*, *C*, *I*, *J*
Photosystem II	*psbA*, *B*, *C*, *D*, *E*, *F*, *H*, *I*, *J*, *K*, *L*, *M*, *N*, *T*, *Z*
Cytochrome b6/f	*petA*, *B* ^(1)^, *D* ^(1)^, *G*, *L*, *N*
ATP synthase	*atpA*, *B*, *E*, *F* ^(1)^, *H*, *I*
Rubisco	*rbcL*
NADH oxidoreductase	*ndhA* ^(1)^, *B* ^(1,^^3)^, *C*, *D*, *E*, *F*, *G*, *H*, *I*, *J*, *K*
Large subunit ribosomal proteins	*rpl2* ^(1,3)^, *14*, *16* ^(^^1)^, *20*, *22*, *23* ^(3)^, *32*, *33*, *36*
Small subunit ribosomal proteins	*rps2*, *3*, *4*, *73* ^(3)^, *8*, *11*, *12* ^(2–4)^, *14*, *15*, *16*, *18*, *19*
RNA polymerase	*rpoA*, *B*, *C1* ^(1)^, *C2*
Unknown function protein-coding gene	*ycf1* ^(3)^, *2* ^(3)^, *3* ^(2)^, *4*
Other genes	*accD*, *ccsA*, *cemA* ^(5)^, *clpP* ^(2)^, *matK*
Ribosomal RNAs	*rrn16* ^(3)^, *23* ^(3)^, *4.5* ^(3)^, *5* ^(3)^
Transfer RNAs	*trnA-UGC* ^(1,3)^, *trnC-GCA*, *trnD-GUC*, *trnE-UUC*, *trnF-GAA*, *trnG-UCC* ^(1)^, *trnG-GCC*, *trnH-GUG*, *trnI-CAU* ^(3)^, *trnI-GAU* ^(1,3)^, *trnK-UUU1* ^(3)^, *trnL-UAA1* ^(3)^, *trnL-UAG*, *trnL-CAA* ^(3)^, *trnM-CAU*, *trnfM-CAU*, *trnN-GUU* ^(3)^, *trnP-UGG*, *trnQ-UUG*, *trnR-ACG* ^(3)^, *trnR-UCU*, *trnS-GCU*, *trnS-GGA*, *trnS-UGA*, *trnT-GGU*, *trnT-UGU*, *trnV-UAC* ^(1)^, *trnV-GAC* ^(3)^, *trnW-CCA*, *trnY-GUA*

^(1)^ Gene containing a single intron; ^(2)^ gene containing two introns; ^(3)^ two gene copies in inverted repeats (IRs); ^(4)^ trans-splicing gene; ^(5)^ pseudogene in the chloroplast (CP) genome of *Fritillaria ussuriensis*.

## Supplementary Materials

Supplementary Materials are available online.

## Abbreviations

CP	Chloroplast
LSC	Large Single Copy
SSC	Small Single Copy
IR	Inverted Repeat
tRNA	Transfer RNA
rRNA	Ribosomal RNA
NGS	Next-Generation Sequencing
KIOM	Korea Institute of Oriental Medicine
ML	Maximum Likelihood
MP	Maximum Parsimony
WGS	Whole-Genome Sequencing
IRb	Inverted Repeat b
SSRs	Simple Sequence Repeats
SNPs	Single-Nucleotide Polymorphisms

## References

[1] Day P.D., Berger M., Hill L., Fay M.F., Leitch A.R., Leitch I.J., Kelly L.J. (2014). Evolutionary relationships in the medicinally important genus *Fritillaria* L. (liliaceae). Mol. Phylogenet. Evol..

[2] Xingi C., Mordak H. (2000). Tulipa L.. Flora China Flagellariaceae Marantaceae.

[3] The State Pharmacopoeia Commission of the People’s Republic of China (2010). Pharmacopoeia of the People’s Republic of China.

[4] Korea Institute of Oriental Medicine (KIOM) (2016). Defining Dictionary for Medicinal Herbs [Korean, ‘hanyak giwon sajeon’]. http://boncho.kiom.re.kr/codex/.

[5] Lee S., Ju Y. (2014). Identification of 11 species of paemo through each original plant and medicines. Korea J. Herbol..

[6] Xiang L., Su Y., Li X., Xue G., Wang Q., Shi J., Wang L., Chen S. (2016). Identification of *Fritillariae* bulbus from adulterants using ITS2 regions. Plant Gene.

[7] Xin G.Z., Lam Y.C., Maiwulanjiang M., Chan G.K., Zhu K.Y., Tang W.L., Dong T.T., Shi Z.Q., Li P., Tsim K.W. (2014). Authentication of Bulbus *Fritillariae* Cirrhosae by RAPD-derived DNA markers. Molecules.

[8] Wicke S., Schneeweiss G.M., de Pamphilis C.W., Muller K.F., Quandt D. (2011). The evolution of the plastid chromosome in land plants: Gene content, gene order, gene function. Plant Mol. Biol..

[9] Daniell H., Lin C.S., Yu M., Chang W.J. (2016). Chloroplast genomes: Diversity, evolution, and applications in genetic engineering. Genome Biol..

[10] Yang M., Zhang X., Liu G., Yin Y., Chen K., Yun Q., Zhao D., Al-Mssallem I.S., Yu J. (2010). The complete chloroplast genome sequence of date palm (*Phoenix dactylifera L.*). PLoS ONE.

[11] Shaw J., Lickey E.B., Schilling E.E., Small R.L. (2007). Comparison of whole chloroplast genome sequences to choose noncoding regions for phylogenetic studies in angiosperms: The tortoise and the hare iii. Am. J. Bot..

[12] Wicke S., Muller K.F., de Pamphilis C.W., Quandt D., Wickett N.J., Zhang Y., Renner S.S., Schneeweiss G.M. (2013). Mechanisms of functional and physical genome reduction in photosynthetic and nonphotosynthetic parasitic plants of the Broomrape family. Plant Cell.

[13] Umesono K., Inokuchi H., Ohyama K., Ozeki H. (1984). Nucleotide sequence of *Marchantia polymorpha* chloroplast DNA: A region possibly encoding three trnas and three proteins including a homologue of *E. coli* ribosomal protein S14. Nucleic Acids Res..

[14] Benson D.A., Karsch-Mizrachi I., Lipman D.J., Ostell J., Rapp B.A., Wheeler D.L. (2000). Genbank. Nucleic Acids Res..

[15] Varshney R.K., Nayak S.N., May G.D., Jackson S.A. (2009). Next-generation sequencing technologies and their implications for crop genetics and breeding. Trends Biotechnol..

[16] Dong W., Liu J., Yu J., Wang L., Zhou S. (2012). Highly variable chloroplast markers for evaluating plant phylogeny at low taxonomic levels and for DNA barcoding. PLoS ONE.

[17] Cho K.S., Yun B.K., Yoon Y.H., Hong S.Y., Mekapogu M., Kim K.H., Yang T.J. (2015). Complete chloroplast genome sequence of tartary buckwheat (*Fagopyrum tataricum*) and comparative analysis with common buckwheat (*F. esculentum*). PLoS ONE.

[18] Luo R., Liu B., Xie Y., Li Z., Huang W., Yuan J., He G., Chen Y., Pan Q., Liu Y. (2012). Soapdenovo2: An empirically improved memory-efficient short-read de novo assembler. Gigascience.

[19] Delcher A.L., Salzberg S.L., Phillippy A.M. (2003). Using mummer to identify similar regions in large sequence sets. Curr. Protoc. Bioinform..

[20] Li Q., Li Y., Song J., Xu H., Xu J., Zhu Y., Li X., Gao H., Dong L., Qian J. (2014). High-accuracy de novo assembly and SNP detection of chloroplast genomes using a SMRT circular consensus sequencing strategy. New Phytol..

[21] Wyman S.K., Jansen R.K., Boore J.L. (2004). Automatic annotation of organellar genomes with DOGMA. Bioinformatics.

[22] Lowe T.M., Eddy S.R. (1997). Trnascan-se: A program for improved detection of transfer RNA genes in genomic sequence. Nucleic Acids Res..

[23] Lohse M., Drechsel O., Bock R. (2007). OrganellarGenomeDRAW (OGDRAW): A tool for the easy generation of high-quality custom graphical maps of plastid and mitochondrial genomes. Curr. Genet..

[24] Tamura K., Stecher G., Peterson D., Filipski A., Kumar S. (2013). Mega6: Molecular evolutionary genetics analysis version 6.0. Mol. Biol. Evol..

[25] Frazer K.A., Pachter L., Poliakov A., Rubin E.M., Dubchak I. (2004). Vista: Computational tools for comparative genomics. Nucleic Acids Res..

[26] Thiel T. (2003). Misa—Microsatellite Identification Tool. http://pgrc.ipk-gatersleben.de/misa/.

[27] Benson G. (1999). Tandem repeats finder: A program to analyze DNA sequences. Nucleic Acids Res..

[28] Warburton P.E., Giordano J., Cheung F., Gelfand Y., Benson G. (2004). Inverted repeat structure of the human genome: The X-chromosome contains a preponderance of large, highly homologous inverted repeats that contain testes genes. Genome Res..

[29] Katoh K., Misawa K., Kuma K.I., Miyata T. (2002). Mafft: A novel method for rapid multiple sequence alignment based on fast fourier transform. Nucleic Acids Res..

[30] Hall T.A. (1999). Bioedit: A user-friendly biological sequence alignment editor and analysis program for windows 95/98/NT. Nucleic Acid Symp. Ser..

[31] Librado P., Rozas J. (2009). DnaSP v5: A software for comprehensive analysis of DNA polymorphism data. Bioinformatics.

[32] Qian J., Song J., Gao H., Zhu Y., Xu J., Pang X., Yao H., Sun C., Li X., Li C. (2013). The complete chloroplast genome sequence of the medicinal plant *salvia miltiorrhiza*. PLoS ONE.

[33] Huang Y.Y., Matzke A.J., Matzke M. (2013). Complete sequence and comparative analysis of the chloroplast genome of Coconut palm (*Cocos nucifera*). PLoS ONE.

[34] Raubeson L.A., Peery R., Chumley T.W., Dziubek C., Fourcade H.M., Boore J.L., Jansen R.K. (2007). Comparative chloroplast genomics: Analyses including new sequences from the angiosperms *Nuphar advena* and *Ranunculus macranthus*. BMC Genom..

[35] Ye C.Y., Lin Z., Li G., Wang Y.Y., Qiu J., Fu F., Zhang H., Chen L., Ye S., Song W. (2014). *Echinochloa* chloroplast genomes: Insights into the evolution and taxonomic identification of two weedy species. PLoS ONE.

[36] Sanchez-Puerta M.V., Abbona C.C. (2014). The chloroplast genome of *Hyoscyamus niger* and a phylogenetic study of the tribe Hyoscyameae (Solanaceae). PLoS ONE.

[37] Kahlau S., Aspinall S., Gray J.C., Bock R. (2006). Sequence of the Tomato chloroplast DNA and evolutionary comparison of Solanaceous plastid genomes. J. Mol. Evol..

[38] Sasaki T., Yukawa Y., Miyamoto T., Obokata J., Sugiura M. (2003). Identification of RNA editing sites in chloroplast transcripts from the maternal and paternal progenitors of Tobacco (*Nicotiana tabacum*): Comparative analysis shows the involvement of distinct trans-factors for *ndhB* editing. Mol. Biol. Evol..

[39] Gao L., Yi X., Yang Y.X., Su Y.J., Wang T. (2009). Complete chloroplast genome sequence of a tree fern *Alsophila spinulosa*: Insights into evolutionary changes in fern chloroplast genomes. BMC Evol. Biol..

[40] Do H.D., Kim J.S., Kim J.H. (2014). A *trnI_CAU* triplication event in the complete chloroplast genome of *paris verticillata* m.Bieb. (melanthiaceae, liliales). BMC Evol. Biol..

[41] Curci P.L., De Paola D., Danzi D., Vendramin G.G., Sonnante G. (2015). Complete chloroplast genome of the multifunctional crop globe artichoke and comparison with other Asteraceae. PLoS ONE.

[42] Zhang H., Li C., Miao H., Xiong S. (2013). Insights from the complete chloroplast genome into the evolution of *Sesamum indicum* L.. PLoS ONE.

[43] Dong W., Xu C., Li C., Sun J., Zuo Y., Shi S., Cheng T., Guo J., Zhou S. (2015). *Ycf1*, the most promising plastid DNA barcode of land plants. Sci. Rep..

[44] Chen J., Hao Z., Xu H., Yang L., Liu G., Sheng Y., Zheng C., Zheng W., Cheng T., Shi J. (2015). The complete chloroplast genome sequence of the relict woody plant *Metasequoia glyptostroboides* Hu et Cheng. Front. Plant Sci..

[45] Liu Q., Xue Q. (2005). Comparative studies on codon usage pattern of chloroplasts and their host nuclear genes in four plant species. J. Genet..

[46] Wolfe K.H., Li W.-H., Sharp P.M. (1987). Rates of nucleotide substitution vary greatly among plant mitochondrial, chloroplast, and nuclear DNAs. Proc. Natl. Acad. Sci. USA.

[47] Kim K.-J., Lee H.-L. (2004). Complete chloroplast genome sequences from Korean ginseng (*panax schinseng* Nees) and comparative analysis of sequence evolution among 17 vascular plants. DNA Res..

[48] Park I., Kim J., Lee J., Kim S., Cho O., Yang K., Ahn J., Nahm S., Kim H. (2013). Development of SSR markers by next-generation sequencing of Korean landraces of chamoe (*Cucumis melo* var. makuwa). Mol. Bio. Rep..

[49] Zalapa J.E., Cuevas H., Zhu H., Steffan S., Senalik D., Zeldin E., McCown B., Harbut R., Simon P. (2012). Using next-generation sequencing approaches to isolate simple sequence repeat (SSR) loci in the plant sciences. Am. J. Bot..

[50] Wang R.J., Cheng C.L., Chang C.C., Wu C.L., Su T.M., Chaw S.M. (2008). Dynamics and evolution of the inverted repeat-large single copy junctions in the chloroplast genomes of monocots. BMC Evol. Biol..

[51] Redwan R.M., Saidin A., Kumar S.V. (2015). Complete chloroplast genome sequence of MD-2 pineapple and its comparative analysis among nine other plants from the subclass Commelinidae. BMC Plant Biol..

[52] Song Y., Dong W., Liu B., Xu C., Yao X., Gao J., Corlett R.T. (2015). Comparative analysis of complete chloroplast genome sequences of two tropical trees *Machilus yunnanensis* and *Machilus balansae* in the family Lauraceae. Front. Plant Sci..

[53] Jansen R.K., Cai Z., Raubeson L.A., Daniell H., Depamphilis C.W., Leebens-Mack J., Muller K.F., Guisinger-Bellian M., Haberle R.C., Hansen A.K. (2007). Analysis of 81 genes from 64 plastid genomes resolves relationships in angiosperms and identifies genome-scale evolutionary patterns. Proc. Natl. Acad. Sci. USA.

[54] Moore M.J., Bell C.D., Soltis P.S., Soltis D.E. (2007). Using plastid genome-scale data to resolve enigmatic relationships among basal angiosperms. Proc. Natl. Acad. Sci. USA.

[55] Lin C.S., Chen J.J., Huang Y.T., Chan M.T., Daniell H., Chang W.J., Hsu C.T., Liao D.C., Wu F.H., Lin S.Y. (2015). The location and translocation of *ndh* genes of chloroplast origin in the Orchidaceae family. Sci. Rep..

[56] Moore M.J., Dhingra A., Soltis P.S., Shaw R., Farmerie W.G., Folta K.M., Soltis D.E. (2006). Rapid and accurate pyrosequencing of angiosperm plastid genomes. BMC Plant Biol..

[57] Kim K., Lee S.C., Lee J., Lee H.O., Joh H.J., Kim N.H., Park H.S., Yang T.J. (2015). Comprehensive survey of genetic diversity in chloroplast genomes and 45s nrDNAs within *panax ginseng* species. PLoS ONE.

[58] Garaycochea S., Speranza P., Alvarez-Valin F. (2015). A strategy to recover a high-quality, complete plastid sequence from low-coverage whole-genome sequencing. Appl. Plant Sci..

[59] Saha M.C., Cooper J.D., Mian M.A., Chekhovskiy K., May G.D. (2006). Tall fescue genomic SSR markers: Development and transferability across multiple grass species. Theor. Appl. Genet..

[60] Yap J.Y., Rohner T., Greenfield A., Van Der Merwe M., McPherson H., Glenn W., Kornfeld G., Marendy E., Pan A.Y., Wilton A. (2015). Complete chloroplast genome of the Wollemi pine (*Wollemia nobilis*): Structure and evolution. PLoS ONE.

[61] Ni L., Zhao Z., Dorje G., Ma M. (2016). The complete chloroplast genome of Ye-Xing-Ba (*Scrophularia dentata*; Scrophulariaceae), an alpine Tibetan herb. PLoS ONE.

[62] Kong W., Yang J. (2016). The complete chloroplast genome sequence of *Morus mongolica* and a comparative analysis within the Fabidae clade. Curr. Genet..

[63] Zhang Y., Du L., Liu A., Chen J., Wu L., Hu W., Zhang W., Kim K., Lee S.C., Yang T.J. (2016). The complete chloroplast genome sequences of five *Epimedium* species: Lights into phylogenetic and taxonomic analyses. Front. Plant Sci..

[64] Arif I.A., Bakir M.A., Khan H.A., Al Farhan A.H., Al Homaidan A.A., Bahkali A.H., Sadoon M.A., Shobrak M. (2010). A brief review of molecular techniques to assess plant diversity. Int. J. Mol. Sci..

[65] Vieira M.L.C., Santini L., Diniz A.L., Munhoz C.D.F. (2016). Microsatellite markers: What they mean and why they are so useful. Genet. Mol. Biol..

[66] Moose S.P., Mumm R.H. (2008). Molecular plant breeding as the foundation for 21st century crop improvement. Plant Physiol..

[67] Yang J.B., Tang M., Li H.T., Zhang Z.R., Li D.Z. (2013). Complete chloroplast genome of the genus *Cymbidium*: Lights into the species identification, phylogenetic implications and population genetic analyses. BMC Evol. Biol..

[68] Khakhlova O., Bock R. (2006). Elimination of deleterious mutations in plastid genomes by gene conversion. Plant J..

[69] Nie X., Lv S., Zhang Y., Du X., Wang L., Biradar S.S., Tan X., Wan F., Weining S. (2012). Complete chloroplast genome sequence of a major invasive species, Crofton weed (*Ageratina adenophora*). PLoS ONE.

[70] Jheng C.F., Chen T.C., Lin J.Y., Chen T.C., Wu W.L., Chang C.C. (2012). The comparative chloroplast genomic analysis of photosynthetic orchids and developing DNA markers to distinguish *Phalaenopsis* orchids. Plant Sci..

[71] Yang Y., Zhou T., Duan D., Yang J., Feng L., Zhao G. (2016). Comparative analysis of the complete chloroplast genomes of five *Quercus* species. Front. Plant Sci..

[72] Li Y., Li Q., Li X., Song J., Sun C. (2016). Complete chloroplast genome sequence of *Fritillaria unibracteata* var. *wabuensis* based on SMRT sequencing technology. Mitochondrial DNA.

[73] Li X., Yang Y., Henry R.J., Rossetto M., Wang Y., Chen S. (2015). Plant DNA barcoding: From gene to genome. Biol. Rev. Camb. Philos. Soc..

