# Supplementary material for: The Complete Chloroplast Genome Sequences of Fritillaria ussuriensis Maxim. and Fritillaria cirrhosa D. Don, and Comparative Analysis with Other Fritillaria Species
Source: Molecules. 2017 Jun 13;22(6):982. doi: 10.3390/molecules22060982 (PMC6152782; doi:10.3390/molecules22060982)
Supplement: Supplementary file 1 [file molecules-22-00982-s001.pdf]

molecules

Supplementary Materials: The Complete Chloroplast Genome Sequences of *Fritillaria ussuriensis* Maxim. and *Fritillaria cirrhosa* D.Don, and Comparative Analysis with Other *Fritillaria* Species

Inkyu Park, Wook-Jin Kim, Sang-Min Yeo, Goya Choi, Young-Min Kang, Renzhe Piao, Byeong-Cheol Moon*

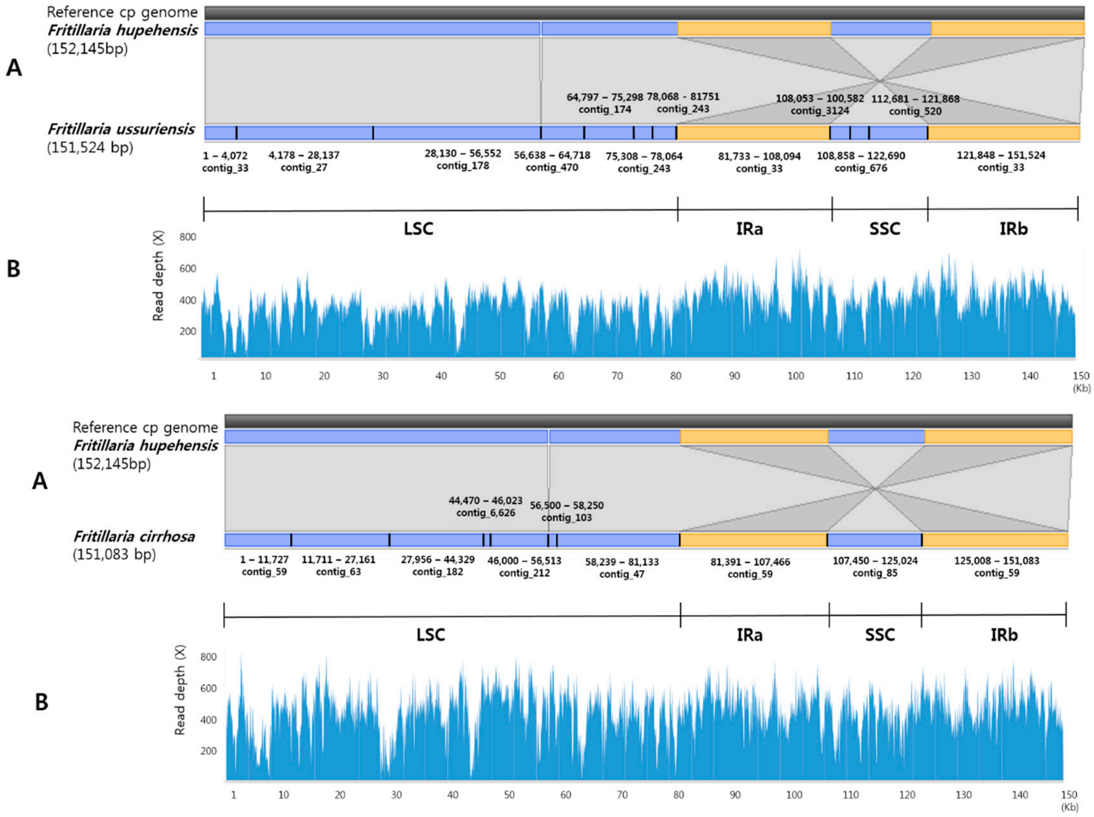

Figure S1 Assembly of the complete chloroplast genome sequences of *Fritillaria* species a. Our assembly result validated to reference of *Fritillaria hupehensis* (GenBank accession NC_024736) using BLASTZ analysis. Alignment of initial contigs was represented on the complete cp genome. The matching contigs positon are denoted at the complete genome sequence. Reference genome and assembly are compared to corresponding regions. b. Mapping of Paired - end reads on the completed cp genome of *Fritillaria* species are represented.

Table S1 Raw read and trimmed read data

Scientific name	Total reads	Total raw bases	Trimmed reads	(%)	Trimmed bases	(%)
Fritillaria ussuriensis	72,445,104	10,893,057,091	40,109,727	55.37	5,045,153,661	46.32
Fritillaria cirrhosa	66,247,268	9,961,547,618	36,032,152	54.39	4,511,872,088	45.29

Table S2 *Fritillaria cp genome assembly information*

Scientific name	Aligned reads #	Coverage (x)	Genome length (bp)	# of contig
Fritillaria ussuriensis	316,479	256.63	151,524	10
Fritillaria cirrhosa	553,582	452.84	151,083	8

Table S3 Base composition of two *Fritillaria* chloroplast genomes

Fritillaria ussuriensis	**A (%)**	**T (%)**	**G (%)**	**C (%)**	**length (bp)**
LSC	32.0	33.3	16.9	17.8	81,732
SSC	34.4	35.0	14.4	16.3	17,114
IR	28.8	28.8	21.2	21.2	52,678
total	31.1	31.9	18.1	18.8	151,524
CDS	31.1	31.5	20.0	17.5	78,951
First position	31.2	24	26.5	18.4	26,317
Second position	29.6	32	17.7	20.2	26,317
Third position	32.3	38	15.8	13.8	26,317
Fritillaria cirrhosa	**A (%)**	**T (%)**	**G (%)**	**C (%)**	**length (bp)**
LSC	31.9	33.3	16.9	17.9	81,390
SSC	34.5	35.1	14.4	16.1	17,537
IR	28.7	28.7	21.3	21.3	52,156
total	31.1	31.9	18.1	18.8	151,083
CDS	31.1	31.5	19.9	17.4	79,835
First position	30.2	29	22.7	18.1	26,612
Second position	31.1	32	18.9	18.1	26,612
Third position	32.1	34	18.2	16.2	26,611

Table S4 Genes with introns in *Fritillaria* chloroplast genomes and the lengths of the exons and introns

Fritillaria ussuriensis						*Fritillaria cirrhosa*					
**Gene**	**exon I**	**intron I**	**exon II**	**intron II**	**exon III**	**Gene**	**exon I**	**intron I**	**exon II**	**intron II**	**exon III**
trnk-UUU	37	2613	35			trnk-UUU	37	2562	35		
rps16	39	882	210			rps16	39	883	210		
trnG-UCC	23	701	37			trnG-UCC	37	697	23		
atpF	144	791	411			atpF	144	786	411		

rpoC1	432	770	1623			rpoC1	432	779	1623		
ycf3	126	733	228	711	159	ycf3	126	742	228	711	159
trnL-UAA	35	533	50			trnL-UAA	35	535	50		
trnV-UAC	39	604	37			trnV-UAC	39	605	37		
rps12*	114	-	232	-	26	rps12	114	-	232	-	26
clpP	71	759	292	593	252	clpP	71	773	292	591	252
petB	6	829	642			petB	6	826	642		
petD	6	772	642			petD	6	761	498		
rpl16	9	996	411			rpl16	9	991	411		
rpl2	393	674	432			rpl2	393	674	432		
ndhB	810	616	756			ndhB	810	617	756		
trnI-GAU	42	933	35			trnI-GAU	42	934	35		
trnA-UGC	38	812	35			trnA-UGC	38	812	35		
ndhA	552	1036	540			ndhA	552	1039	540		

*rps12 gene is trans-splicing gene.

Table S5 Codon-anticodon recognition pattern and codon usage in *Fritillaria* genomes

Amino acid	Codon	*Fritillaria ussuriensis*		*Fritillaria cirrhosa*		tRNA
		Count	RSCU*	Count	RSCU	
Phe	UUU(F)	965	1.29	988	1.23	
Phe	UUC(F)	530	0.71	622	0.77	trnF-GAA
Leu	UUA(L)	904	2	763	1.7	trnL-UAA
Leu	UUG(L)	520	1.15	580	1.29	trnL-CAA
Leu	CUU(L)	573	1.27	513	1.14	
Leu	CUC(L)	183	0.41	249	0.55	
Leu	CUA(L)	364	0.81	390	0.87	trnL-UAG
Leu	CUG(L)	165	0.37	205	0.46	
lle	AUU(I)	1094	1.42	1039	1.36	
lle	AUC(I)	450	0.59	553	0.73	trnI-GAU
lle	AUA(I)	763	0.99	695	0.91	trnI-CAU trn(f)M-CAU trnM-CAU
Met	AUG(M)	638	1	560	1	trnM-CAU
Val	GUU(V)	510	1.45	485	1.43	
Val	GUC(V)	186	0.53	208	0.61	trnV-GAC
Val	GUA(V)	512	1.46	447	1.32	trnV-UAC
Val	GUG(V)	195	0.56	217	0.64	
Ser	UCU(S)	576	1.68	522	1.49	
Ser	UCC(S)	325	0.95	331	0.94	trnS-GGA
Ser	UCA(S)	441	1.29	467	1.33	trnS-UGA
Ser	UCG(S)	179	0.52	254	0.73	
Pro	CCU(P)	406	1.52	334	1.34	
Pro	CCC(P)	224	0.84	208	0.83	trnS-GCU
Pro	CCA(P)	317	1.19	312	1.25	
Pro	CCG(P)	123	0.46	144	0.58	
Thr	ACU(T)	531	1.58	390	1.31	trnP-UGG
Thr	ACC(T)	246	0.73	238	0.8	
Thr	ACA(T)	416	1.24	394	1.32	
Thr	ACG(T)	153	0.45	173	0.58	trnT-GGU
Ala	GCU(A)	603	1.75	433	1.65	trnT-UGU
Ala	GCC(A)	220	0.64	183	0.7	
Ala	GCA(A)	403	1.17	305	1.17	
Ala	GCG(A)	155	0.45	126	0.48	trnG-UCC
Tyr	UAU(Y)	826	1.64	827	1.45	trnA-UGC
Tyr	UAC(Y)	181	0.36	317	0.55	
Stop	UAA(*)	45	1.61	438	1.17	
Stop	UAG(*)	20	0.71	318	0.85	trnY-GUA
His	CAU(H)	501	1.58	483	1.44	
His	CAC(H)	132	0.42	189	0.56	
Gln	CAA(Q)	692	1.5	682	1.43	
Gln	CAG(Q)	228	0.5	271	0.57	
Asn	AAU(N)	1020	1.57	892	1.46	trnH-GUG
Asn	AAC(N)	280	0.43	332	0.54	trnQ-UUG
Lys	AAA(K)	1048	1.46	1026	1.4	
Lys	AAG(K)	389	0.54	441	0.6	

Asp	GAU(D)	861	1.61	646	1.51	trnN-GUU
Asp	GAC(D)	211	0.39	211	0.49	trnK-UUU
Glu	GAA(E)	1021	1.49	851	1.43	
Glu	GAG(E)	346	0.51	341	0.57	
Cys	UGU(C)	231	1.5	290	1.25	trnD-GUC
Cys	UGC(C)	77	0.5	173	0.75	trnE-UUC
Stop	UGA(*)	19	0.68	367	0.98	
Trp	UGG(W)	452	1	469	1	
Arg	CGU(R)	351	1.33	248	0.92	trnC-GCA
Arg	CGC(R)	96	0.36	106	0.39	trnW-CCA
Arg	CGA(R)	352	1.34	309	1.15	trnR-ACG
Arg	CGG(R)	131	0.5	165	0.61	
Ser	AGU(S)	435	1.27	355	1.01	
Ser	AGC(S)	99	0.29	173	0.49	
Arg	AGA(R)	503	1.91	534	1.99	trnR-UCU
Arg	AGG(R)	147	0.56	249	0.93	
Gly	GGU(G)	546	1.25	471	1.19	
Gly	GGC(G)	186	0.42	211	0.53	trnG-GCC
Gly	GGA(G)	706	1.61	558	1.41	trnG-UCC
Gly	GGG(G)	315	0.72	340	0.86	

*RSCU – Relative synonymous codon usage

Table S6 Distribution of tandem repeats in two *Fritillaria* species cp genomes

F. ussuriensis	position	Repeat unit length (bp)	Repeat unit sequecne	Repeat numbers	Region
1	Intron (rps16, rps16)	13	TTATAAATTTATA	2	LSC
2	IGS (rps16, trnQ-UUG)	14	TATAATATTAAATA	3	LSC
3	IGS (trnR-UCU, atpA)	13	AATTATTATTCTT	2	LSC
4	IGS (trnT-UGU, trnL-UAA)	54	TAATTATTAATTATCTCTAAAATAAATTATAT AATAAATTATATTATATAATAT	2	LSC
5	IGS (trnF-GAA, ndhJ)	13	CATATATATCTAA	2	LSC
6	IGS (rbcL, accD)	15	TTATTTGTATTGTTTA	2	LSC
7	IGS (psaJ, rpl33)	13	TTATTTTTTTTTT	2	LSC
8	IGS (petD, rpoA)	33	TAAATCCATTCTATAATATATATATATAAATTA TA	2	LSC
9	CDS (rps11)	24	TACGTCCATTCTTACGTGAACCAA	2	LSC
10	IGS (rps8, rpl14)	24	TTTAATAAAAAATAATTAAATTTA	2	LSC
11	IGS (rpl22, rps19)	1	AAAAAAAAAAAAAAAAAAAAAAAAA	25	LSC
12	CDS (ycf2)	21	CTTTTTGTCCAAGTCACTTCC	2	IR
13	CDS (ycf2)	24	TGACGATATCGATTTTGATGATAG	2	IR
14	IGS (psaC, ndhE)	47	TATTATTATATAATTTATATAATGAAAATCA ATGATTATATAATGAA	2	SSC
15	CDS (ycf1)	15	CATAATTAATATATT	2	SSC
F. cirrhosa					
1	IGS (matK, trnK-UUU)	14	AATGATACATAGTG	2	LSC
2	IGS (trnS-GCU, trnG-UCC)	17	TATACATATATTAAATA	2	LSC
3	IGS (trnG-UCC, trnR-UCU)	47	AAAGAATTTGAAAAGTTAGGAATGAAAAGC GTCCATTGTCTAATGGA	2	LSC
4	IGS (psbM, trnD-GUC)	1	AAAAAAAAAAAAAAAAAAAAAAAAAAA	27	LSC
5	IGS (trnT-GGU, psbD)	22	ATACATATATAACATATATAAC	3	LSC
6	IGS (trnT-UGU, trnL-UAA)	12	TATATTATATAA	2	LSC
7	IGS (trnT-UGU, trnL-UAA)	21	TAAGATAAGAATAAAGATCAT	2	LSC
8	IGS (trnF-GAA, ndhJ)	13	CATATATATCTAA	2	LSC
9	IGS (rbcL, accD)	14	TAAATATATAAGTA	2	LSC
10	CDS (ycf2)	24	TGACGATATCGATTTTGATGATAG	3	IR
11	CDS (ycf2)	24	ATATCGTCACTATCATCAATATCG	3	IR
12	CDS (ycf1)	15	CATAATTAATATATT	2	SSC
13	CDS (ycf1)	15	ATTATTATTACTAGT	2	SSC

Table S7 Palindromic repeats in two *Fritillaria* cp genomes

	Position	Repeat unit length (bp)	Repeat units sequences	Repeat numbers	Loop (bp)	Region
F. ussuriensis	IGS (accD, psaI)	30	ATAATATATCATATTTGAATCTTAAATATTA	2	4	LSC
	IGS (petD, poA)	29	TAAATCCATTCTATAATATATATATAAAT	2	1	LSC
	IGS (ccsA, dhD)	28	ATGTATCTCGAGTTTTTGCGAACCATTT	2	13	SSC
	IGS (rps15, ycf1)	28	TTTTATTCTATCGAAATCCCATTTTTAT	2	7	SSC
F. cirrhosa	IGS (petN, psbM)	36	AATCATGAGATAGTGTGTAGAAAAAACTATAGATAT	2	1	LSC
	IGS (accD, psaI)	31	ATAATATATCATATTTGAATCTTAAATATTA	2	4	LSC
	IGS (petD, rpoA)	25	TAAATCCATTCTATAATATATAAAT	2	5	LSC
	IGS (rpl32, trnL-UAG)	24	TTTTTCTTTATCAATTAAATATTC	2	6	SSC
	IGS (ccsA, ndhD)	28	ATGTATCTCGAGTTTTTGCGAACCATTT	2	13	SSC
	IGS (rps15, ycf1)	25	TTTTATTCTATCGAAATCCCATTTT	2	12	SSC

[a] IGS; Intergenic sequence [b] LSC; Large single copy, IR; Inverted repeat region, SSC; Small single copy

Table S8 Polymorphic SSRs between *F. ussuriensis* and *F. cirrhosa* cp genomes

No.	Location	Region	Motif	Repeat number	
				F. ussuriensis	***F. cirrhosa***
1	psbA-trnK	IGS	T	13	17
2	trnK-rps16	IGS	A	17	8
3	trnK-rps16	IGS	T	22	11
4	rps16-trnQ	IGS	T	10	11
5	trnS-trnG	IGS	A	11	14
6	trnS-trnG	IGS	T	11	10
7	atpF-atpF	Inton	A	14	15
8	atpH-atpI	IGS	T	12	9
9	atpH-atpI	IGS	A	11	10
10	rps2-rpoC2	IGS	T	11	16
11	trnD-trnY	IGS	T	13	14
12	trnE-trnT	IGS	T	14	18
13	trnT-psbD	IGS	A	11	14
14	trnT-psbD	IGS	AT	8	10
15	psbZ-trnG	IGS	A	24	12
16	ycf3-ycf3	intron	A	17	12
17	ycf3-ycf3	intron	A	9	8
18	trnS-rps4	IGS	A	10	12
19	rps4-trnT	IGS	T	9	8

20	trnT-trnL	IGS	A	21	17
21	trnT-trnL	IGS	A	9	17
22	trnL-trnL	IGS	AT	10	12
23	trnL-trnF	IGS	T	13	18
24	trnF-ndhJ	IGS	T	9	8
25	accD-psaI	IGS	A	10	15
26	accD-psaI	IGS	A	17	8
27	accD-psaI	IGS	A	9	12
28	ycf4-petA	IGS	A	17	20
29	petA-psbJ	IGS	AT	8	10
30	psbE-petL	IGS	T	13	14
31	trnW-trnP	IGS	A	13	15
32	trnP-psaJ	IGS	T	11	8
33	psaJ-rpl33	IGS	A	11	16
34	clpP-clpP	intron	T	16	15
35	clpP-clpP	intron	A	11	13
36	clpP-clpP	intron	A	9	8
37	clpP-clpP	intron	T	10	11
38	clpP-psbB	IGS	A	10	13
39	petD-rpoA	IGS	AT	10	14
40	rpl16-rpl16	intron	T	17	14
41	rpl16-rps3	IGS	T	18	15
42	rps3	exon	T	11	8
43	rpl22-rps19	IGS	A	25	23
44	ndhF-rpl32	IGS	A	8	10
45	rpl32-trnL	IGS	T	13	11
46	ndhG-ndhI	IGS	AAAT	12	16
47	ndhA-ndhA	intron	A	13	12
48	ndhH-rps15	IGS	A	8	9
